# Bot or Not? Detecting and Managing Participant Deception When Conducting Digital Research Remotely: Case Study of a Randomized Controlled Trial

**DOI:** 10.2196/46523

**Published:** 2023-09-14

**Authors:** Gemma Loebenberg, Melissa Oldham, Jamie Brown, Larisa Dinu, Susan Michie, Matt Field, Felix Greaves, Claire Garnett

**Affiliations:** 1 UCL Tobacco and Alcohol Research Group University College London London United Kingdom; 2 Clinical Educational and Health Psychology University College London London United Kingdom; 3 Department of Psychology University of Sheffield Sheffield United Kingdom; 4 Department of Primary Care and Public Health Imperial College London London United Kingdom

**Keywords:** artificial intelligence, false information, mHealth applications, participant deception, participant, recruit, research subject, web-based studies

## Abstract

**Background:**

Evaluating digital interventions using remote methods enables the recruitment of large numbers of participants relatively conveniently and cheaply compared with in-person methods. However, conducting research remotely based on participant self-report with little verification is open to automated “bots” and participant deception.

**Objective:**

This paper uses a case study of a remotely conducted trial of an alcohol reduction app to highlight and discuss (1) the issues with participant deception affecting remote research trials with financial compensation; and (2) the importance of rigorous data management to detect and address these issues.

**Methods:**

We recruited participants on the internet from July 2020 to March 2022 for a randomized controlled trial (n=5602) evaluating the effectiveness of an alcohol reduction app, Drink Less. Follow-up occurred at 3 time points, with financial compensation offered (up to £36 [US $39.23]). Address authentication and telephone verification were used to detect 2 kinds of deception: “bots,” that is, automated responses generated in clusters; and manual participant deception, that is, participants providing false information.

**Results:**

Of the 1142 participants who enrolled in the first 2 months of recruitment, 75.6% (n=863) of them were identified as bots during data screening. As a result, a CAPTCHA (Completely Automated Public Turing Test to Tell Computers and Humans Apart) was added, and after this, no more bots were identified. Manual participant deception occurred throughout the study. Of the 5956 participants (excluding bots) who enrolled in the study, 298 (5%) were identified as false participants. The extent of this decreased from 110 in November 2020, to a negligible level by February 2022 including a number of months with 0. The decline occurred after we added further screening questions such as attention checks, removed the prominence of financial compensation from social media advertising, and added an additional requirement to provide a mobile phone number for identity verification.

**Conclusions:**

Data management protocols are necessary to detect automated bots and manual participant deception in remotely conducted trials. Bots and manual deception can be minimized by adding a CAPTCHA, attention checks, a requirement to provide a phone number for identity verification, and not prominently advertising financial compensation on social media.

**Trial Registration:**

ISRCTN Number ISRCTN64052601; https://doi.org/10.1186/ISRCTN64052601

## Introduction

Conducting studies remotely using digital technology such as web-based survey tools offers several benefits and was particularly useful during the COVID-19 pandemic, which precluded face-to-face contact for long periods. Remote participation has benefits for both participants and researchers. It is accessible [1] and convenient for participants [2], as they can enroll from anywhere at any time, an option that conventional face-to-face research does not always offer. Similarly, it is more convenient for researchers, as there is the potential for recruiting large numbers of participants quickly and at a low cost. This method of recruitment may also achieve better external validity if digital interventions are being evaluated.

A major disadvantage is that conducting studies remotely tends to rely on participant honesty in self-report, and researchers cannot be sure that the participant is who they say they are; participants have been known to engage in deception to take part in research with financial incentives available [3]. It is relatively simple for people to create multiple email accounts and use other false information, so they can sign up multiple times if they wish. Indeed, it has been noted that those who want to defraud research are able to do so on a larger scale on the internet than would usually be possible with in-person projects [4]. Researchers can verify participants’ eligibility by requiring screenshots of ID or phone calls, though this increases the participant burden and is likely to result in fewer genuine participants enrolling as well as being burdensome for researchers.

This study differentiates between 2 main types of participant deception that can occur in remotely conducted studies and cause significant issues for researchers: bots and manual participant deception. Automated “bots” (short for “robots”) [5] are programmed to perform automated tasks on the internet and can impersonate human users [2]. In this study, they were differentiated by the volume of entries that occurred in a short space of time. Manual participant deception is where individuals provide false information, usually across multiple entries. Automated bots may be created by individuals motivated by causing disruption of the kind which became widespread during the pandemic. For example, hijacking Zoom meetings when large-scale use of the platform increased during the pandemic [6]. Individuals may also have been motivated by gaining the financial compensation available through repeated participation in the trial. Manual participant deception has been described as “professional subjects” [7] who join several studies, or the same study multiple times, in order to create income; such participants may dishonestly claim to meet the inclusion criteria, for example by falsely confirming they have the illness being studied.

This is not a new issue, and previous remote studies have encountered bots and detailed management techniques, such as differentiating between automated strategies embedded into electronic surveys and manual plans during recruitment [8], adding a statement that fraudulent entries would not receive compensation [9], and using dynamic methods to detect fraud that adapt to “bot learning” [10]. Additionally, researchers found that 60.4% of responses to a web-based study (n=478) were likely fraudulent, following publication of a Facebook advertisement where a US $50 gift card compensation was mentioned [11]. However, the COVID-19 pandemic and consequent social distancing measures meant that many studies had to move on the internet, which may have led to a rise in participant deception when compensation was available [9]. When studies are conducted in person, it has been suggested that participants assume more responsibility for their actions [12]. Researchers have warned that a lack of awareness of such fraud and not having procedures in place to mitigate against it risks undermining remotely conducted research [2]. Fraudulent responses can cause problems with data validity [3,8] by introducing random noise into studies, which could impact results and lead to inaccurate conclusions being made. Without appropriate procedures, remotely conducted research could be considered less robust than traditional face-to-face methods. Even when employing fraud detection strategies, there can be issues with participant deception. In 1 remote study, researchers found that 28.7% of their survey responses (n=414) were fraudulent after completing data collection [13]. Studies should have dynamic protocols that can adapt in response to changes in deceit [11], and antideception protocols should be included in grant applications and other associated study paperwork [2]. Participant deception in research is likely to evolve and adapt to strategies intended to mitigate it. It is therefore important to update and share details on the issues that participant deception can create in digital research, detection strategies, and data management procedures.

This paper reports a case study of a remotely conducted randomized controlled trial of an alcohol reduction app, *Drink Less* (the iDEAS trial) [14*,*15], which encountered problems with participation deception from the opening of recruitment during the COVID-19 pandemic, explains how it was identified, managed, and resolved, and provides guidance on how to avoid similar issues in remote web-based research.

## Methods

CONSORT (Consolidated Standards of Reporting Trials) reporting guidelines [16] were used in this paper.

### Trial Context

The iDEAS trial aimed to evaluate the effectiveness of *Drink Less* [14,15], a smartphone app, compared with the National Health Service (NHS) alcohol advice web page, in reducing alcohol consumption among increasing and higher-risk adult drinkers in the United Kingdom. It began recruitment as large parts of the United Kingdom population had recently emerged from a strict lockdown due to the COVID-19 pandemic, with restrictions continuing in varying forms throughout most of the recruitment period [17]. It provided financial compensation (up to £36 [US $39.23] in vouchers) for completing 3 follow-up surveys over a 6-month period.

The original recruitment plan prepandemic was to place posters in NHS Primary Care services, but this has to be moved to web-based media when face-to-face appointments became remote appointments. Because recruitment occurred at this time, when many people were at home, people may have had more motivation and time to engage in deception.

The 21-month trial recruitment period ran from July 13, 2020, to March 31, 2022, with a monthly target of 265 (total recruitment target, n=5562). Minimal advertising on Twitter (a tweet from the University and a promotion from the funder) occurred in July. The study began advertising on Facebook and Google in September 2020 (Figures S1 in Multimedia Appendix 1) and on the NHS website [18] in October 2020 (Multimedia Appendix 2). Advertising in primary care took place in November 2021 (Multimedia Appendix 3), and radio advertising took place in January and February 2022 (see Multimedia Appendix 4 for the advertisement transcript).

After completing a baseline eligibility survey on Qualtrics, participants were randomized to 1 of 2 conditions (either the *Drink Less* app [14*,*15] or the NHS alcohol advice webpage [18]) and received web-based follow-up surveys at 1, 3, and 6 months after randomization. Participants were compensated for their time with web-based gift vouchers that were emailed for completing these follow-up surveys: £6 (US $ 6.54) at 1 and 3 months, and £12 (US $13.08) at 6 months, with an additional £12 (US $13.08) paid if the 6-month follow-up survey was completed within 24 hours (a maximum total of £36 [US $39.23]). At all follow-up stages, participants were emailed the survey link up to 3 times if no response was received. At the 6-month follow-up, after the third email, participants were contacted twice by telephone. If there was still no response to the survey after 18 days, it was sent out by post with a FREEPOST return envelope. After 28 days, if no response was received, a final short postcard with just the primary outcome variable (AUDIT-C) was posted to the participant. Full details are available in the study protocol [19].

**Figure 1:**
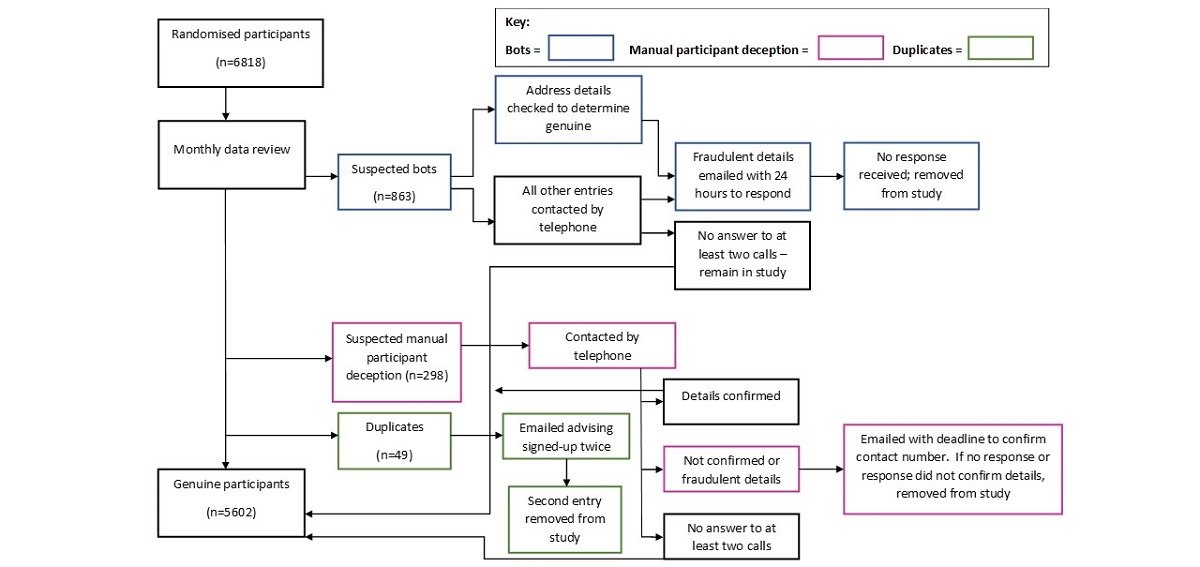
Enrollment decision tree.

From the outset of recruitment, problems were experienced with participant deception. Figure 1 illustrates the process followed for each type of recruitment problem. We now discuss how the 2 main issues, automated bots and manual participant deception, were identified and addressed during the study. Duplicate entries were a much smaller concern, and strategies for dealing with them have been addressed by other authors [13].

### Bot Deception

#### Definition

Bots were identified as (1) an entry enrolling in a cluster (multiple entries in the same hour, with a similar style of email address) who (2) provided postcodes that did not match international street names entered and provided international phone numbers.

#### Identification of the Problem

Within the first 19 days of recruitment, with minimal advertising that was expected to have low reach (a tweet from the university and a promotion from the funder), the monthly recruitment target of 265 participants had been surpassed with 870 randomized participants (15.6% of the overall study target). The anticipated rate of recruitment was based on previous experience (an earlier factorial trial of *Drink Less*, which recruited 355 participants per month). Consequently, the research team reviewed all the data, which revealed some enrollment was arriving in batches, with clusters of “people” joining simultaneously in the early hours of the morning, using non–United Kingdom street names. Bots were distinguishable by either providing a postcode that did not match the first line of the street address given or being unknown at the phone number provided, and by the rate at which entries joined the study, usually at odd hours of the night. At its peak, on July 30, 2020, there were 41 enrollments between 6 and 7 AM, all of whom were classified as automated bots.

#### Management

There were 2 main stages to the management process.

##### Postcode Checks

All randomized participants’ postcodes were checked (enrollments had now risen to n=915) to assess whether they matched with the first line of an address provided by Royal Mail Postcode Finder [20]; 561 suspicious entries were identified. All were emailed with a 24-hour notice of deletion unless they responded (Multimedia Appendix 5); none were confirmed as real, and all were removed from the study.

Different options were considered to avoid future bot responses, including Qualtrics’ fraud detection software options, such as a reporting tool to indicate whether a response is likely to be a bot [21]. However, this would not prevent future additional bot enrollments as it only provides information about whether an entry was considered fraudulent at the end of the survey. A CAPTCHA (Completely Automated Public Turing Test to tell Computers and Humans Apart) was selected as this was a quick remedy to prevent future bots and was added to the baseline survey on Qualtrics on August 11, 2020.

A second round of checks on participants (n=196) were reviewed to identify whether any bots had enrolled (1) since the original checks but before the addition of the CAPTCHA and (2) following its addition. A further 181 suspicious entries were identified as enrolling (in the intervening period between the original checks and the addition of the CAPTCHA).

##### Telephone Checks

To further avoid the likelihood of including fraudulent entries, every participant who enrolled before the CAPTCHA had been added was contacted by phone. A participant was classified as a bot if either (1) the number provided was false or (2) it was confirmed the participant was not known at that number.

To minimize bias in removing participants after randomization, decisions erred on the side of inclusion, and unless there was proof that participants were not real, they remained in the study. For example, a participant remained in the study even if nobody answered the phone after 2 attempts. An additional 121 bots were identified and emailed as before, allowing 24 hours to respond with verified contact information.

### Manual Participant Deception

**Figure 2:**
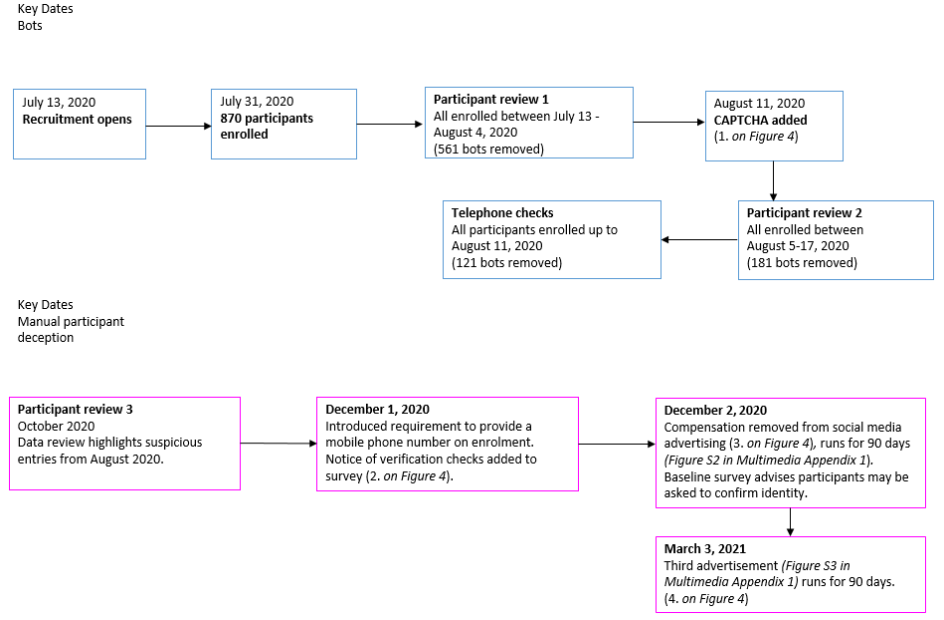
Dates and changes to procedures in response to problems arising. CAPTCHA: Completely Automated Public Turing Test to Tell Computers and Humans Apart.

Monthly data checks from October 2020 (Figure 2) identified a different issue of manual participant deception rather than automated bots.

#### Definition

Manual participant deception was defined as when a participant signed up for the trial and provided false contact information, confirmed by verification checks. This comprised either (1) invalid contact numbers where participants were not known, (2) an address where the postcode did not match the first line of the address, or (3) the same landline number provided by multiple respondents with different geographical postal addresses (where the likelihood of the number being shared was slim).

#### Identification

Manual participant deception was first identified during October 2020, relating to a participant randomized in August, with postcode checks [20] then undertaken for all participants enrolled from August 2020 onward. Any participants suspected to be false based on the above criteria were emailed with 24 hours to respond (Textbox S1 in Multimedia Appendix 6).

This issue was distinctive mainly through the use of landline phone numbers or the addresses of large companies. Examples included web-based estate agents, London hotels and restaurants, charities, and even funeral homes. As described, part of the procedure was to verify all addresses using Royal Mail’s website [20], and business addresses were easily identified. Landline numbers were likely used because they are widely available on the internet for large businesses, and it is uncommon in the United Kingdom for an individual to have access to multiple mobile phone numbers. Consequently, it became a trial requirement to provide a mobile phone number for identity verification, as it was considered harder to provide a false mobile phone number. Repeated use of landline numbers made suspicious entries easier to identify and contact.

#### Management

The management procedure is shown in Figure 1. Initially, individuals were given 24 hours to respond, but this was extended to give participants 72 hours to respond, following feedback from genuine participants the team spoke with that the deadline was too short (Textbox S2 in Multimedia Appendix 6).

In an attempt to mitigate against participant deception, the mention of the financial compensation was removed from social media advertising (Figure S2 in Multimedia Appendix 1), and the Qualtrics baseline survey was updated to advise participants that they may be asked to confirm their identity. In order to be objective and include as many participants as possible, business addresses were not automatically considered suspicious unless accompanied by a landline phone number; this was treated as suspicious and managed by attempting to call the participant (there were only a few cases where a participant was known at a business address). If a mobile number was given, participants were emailed to check that they wanted follow-up surveys to be sent to a business address rather than a home address. If they did not respond, we attempted to call them on up to 2 occasions. If we did not reach them to confirm their details, they remained in the study; if the person enrolled was not known at that number, they were emailed advising that we had been unable to reach them on the number provided (Textbox S3 in Multimedia Appendix 6). If no response was received or details were not confirmed, they were removed from the study.

When we spoke to a genuine participant (whose name matched the person we reached on the phone), we explained we had called to confirm the details provided, and they remained in the study. From December 2020 onward, a percentage of participants were called at random each month to verify their details.

In March 2021, we amended the social media advertising to reinclude the mention of the financial compensation but reduce the prominence of the financial compensation available, in addition to targeting Facebook advertising so it could only be viewed by males to try and achieve a more representative sample. This compromise was a balance between reducing the rate of participant deception and minimizing the impact on genuine recruitment, leading to a fall in the rate of manual participant deception. The advertising had specific parameters in terms of who it was displayed to at this time, which reduced the audience it was displayed to on Facebook. This second advertisement, which ran on social media for 90 days, was replaced by a final edit (Figure S3 in Multimedia Appendix 1), and at other times the study was subsequently advertised on social media. There was no substantial difference in the number of “clicks” received over the course of the 2nd and 3rd advertisement display periods, but fewer participants enrolled when there were no financial incentives prominent in the advertisement. Of note, we did not have to employ this method for our physical or radio advertising (Multimedia Appendix 3 and 4).

The other method we employed was an additional attention check on the baseline survey. One had been placed in the survey initially, a question: “Just checking that you are a human, please select ‘weekly’ as your answer to this question”. If they did not, they were screened out (62/7300, 0.8%). To further protect against manual deception, in November 2020, a second check was added, asking participants to enter their age, then, after a few further blocks of questions, to enter their age again. If these responses did not match, the participants were screened out (135/7300, 1.85%).

One of the difficulties during this process was balancing the need to recruit large numbers of participants to detect small but meaningful effects while avoiding encouraging many attempts at fraud by making incentives too prominent. We were also mindful of trying to recruit a representative sample while also ensuring that participants were genuine. We were fortunate to have the support of NHS Digital in placing an advertisement (Multimedia Appendix 2*)*; the impact of advertising considerations is discussed further in the related trial methods paper [22].

At each stage, a problem was identified, the core research team discussed the issue and how best to resolve it, and these decisions were checked with the full trial team, the Data Monitoring and Trial Steering Committees. The approach was flexible and reactive, depending on how the problem manifested. The decisions described in the case study were made on the basis of inclusion, with participants included in the study unless we could confirm that there was participant deception. Despite the issues experienced, the iDEAS trial successfully recruited participants to time and target.

### Ethics Approval

Ethical approval for this study was obtained from the UCL Research Ethics Committee (16799/001).

## Results

### Bot Deception

A review of all participants enrolled in the study during the first 2 months of recruitment revealed only 23.4% (204/870) of participants in July and 27.6% (75/272) of participants in August were not automated bots.

In total, 75.6% (863/1142) of sign-ups in this period were classified as bots with no bots identified after the addition of the CAPTCHA.

**Figure 3:**
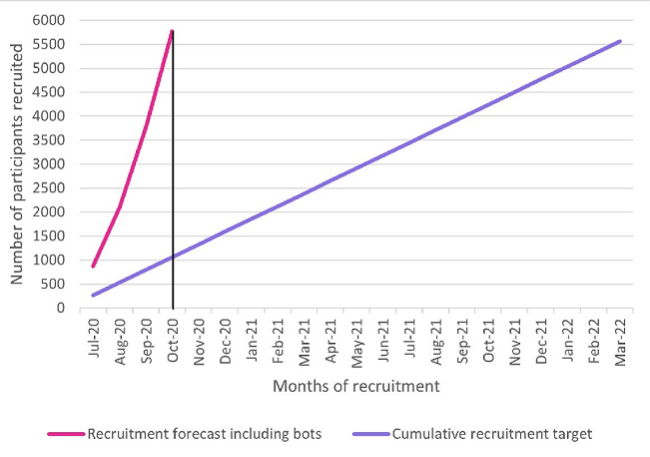
Forecast illustrating disruption to recruitment if bots are undetected.

Figure 3 shows a forecast illustrating the potential disruption to recruitment if the bots were not identified. Based on a rolling average of approximately 48 bots enrolling per day, the recruitment target (n=5562) would have been met 17 months ahead of schedule.

### Manual Participation Deception

**Figure 4:**
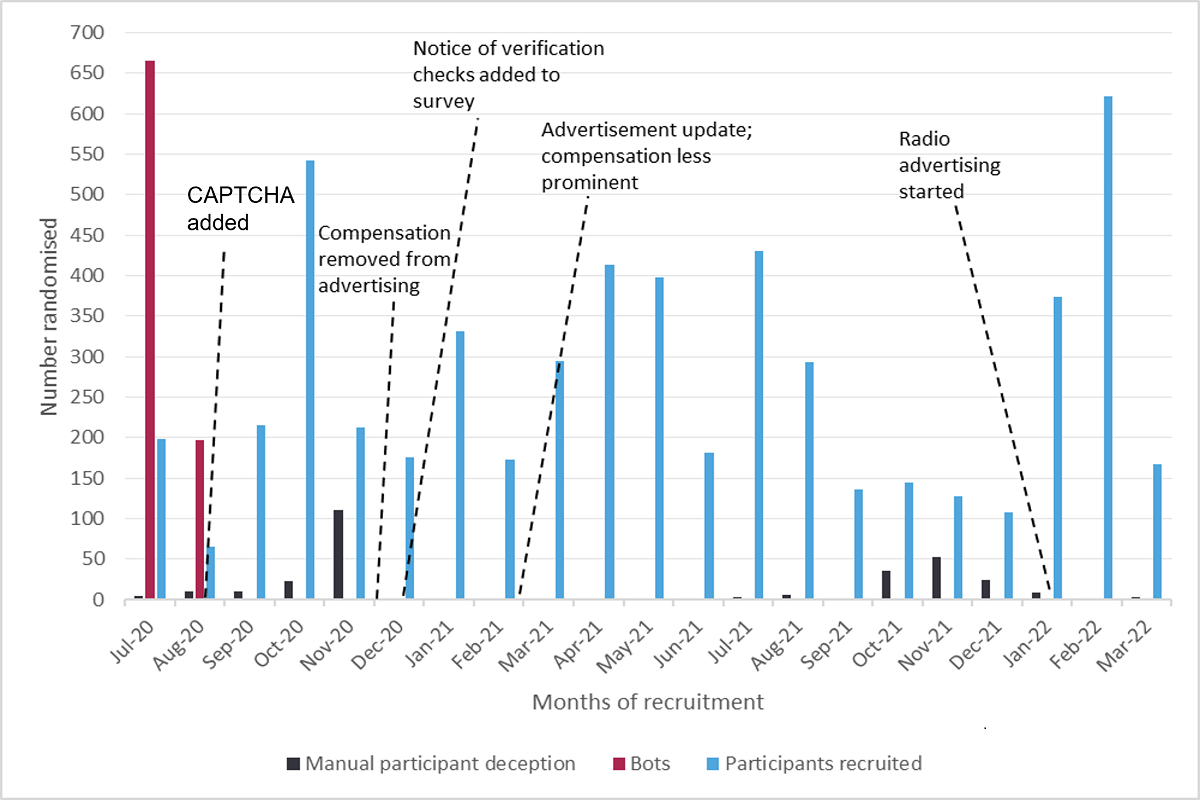
Manual participant deception, bots, and actual recruitment throughout the study. CAPTCHA: Completely Automated Public Turing Test to Tell Computers and Humans Apart.

In total, 4.3% (294/6818) of randomized participants were identified as having engaged in manual participant deception during the entire recruitment period. As illustrated in Figure 4, the prevalence of participant deception fluctuated during recruitment, peaking in November 2020 with 34% (110/324 enrollments) and again in November 2021 with 29.1% (53/182 enrollments) of participants identified as false.

Updating the advertising helped reduce the number of false responses but also reduced the number of genuine participants enrolling. Figure 4 also shows the monthly recruitment rate for the duration of the study, with the horizontal line indicating the monthly target of 265 participants. This fluctuated throughout recruitment and varied according to the promotion methods employed, in addition to the months where the project was heavily targeted by bots or manual participant deception.

Because of the impact on the recruitment of genuine participants, a third version of the advertisement was used, where compensation was mentioned but not given prominence (Figure S3 in Multimedia Appendix 1).

**Figure 5:**
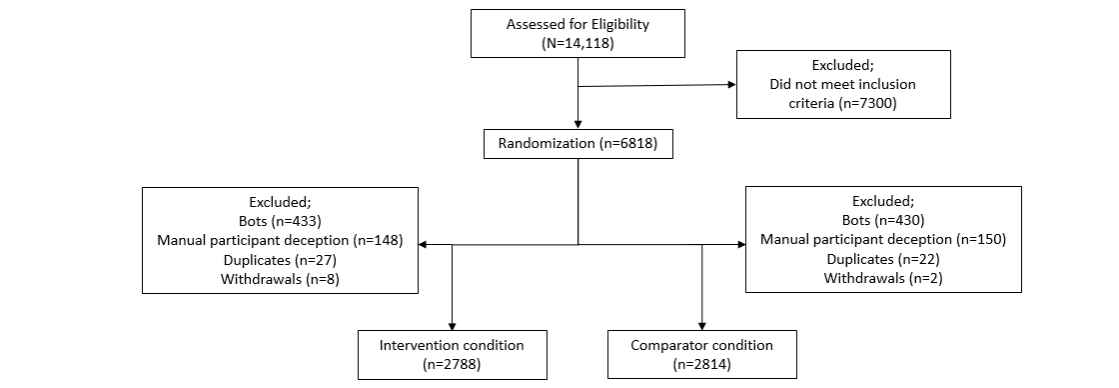
CONSORT (Consolidated Standards of Reporting Trials) diagram of participant numbers and reasons excluded.

The rate of manual participant deception was reduced as a result of further screening questions, diligent checks by the research team, and excluding false participants from the study. This fluctuated throughout the study (Figure 4), but using the processes outlined, we continued to identify and exclude false participants. Figure 5 details the numbers of those excluded and the reasons why.

## Discussion

### Overview

This paper presents a case study on participant deception experienced throughout a remotely conducted large randomized controlled trial. Two types of participant deception were detected: automated bots and manual participant deception. Automated bots were identified based on clusters of enrollments very early in the morning with non–United Kingdom–based addresses. A CAPTCHA was added that resolved the issue, and no additional bots were identified. Manual participant deception was discovered due to incorrect or repetitive information provided, including restaurant and hotel contact details instead of home addresses. Monthly data checks to identify any unexpectedly high recruitment periods, suspicious entries, and contacting participants swiftly helped to mitigate the problem, although it required close monitoring throughout recruitment.

Considering the circumstances under which recruitment took place, some of our experiences and recommendations may not be applicable when conducting research outside of a pandemic; this is an empirical question. While it is likely the pandemic meant some people were more engaged with web-based research, the highest recruiting day with the most suspicious entries occurred with 312 enrollments on July 29, 2020, when no active recruitment or social media advertising was taking place. Such automated bots have the potential to seriously disrupt recruitment and bias the final results. In our trial, we forecast that the target (n=5562) would have been reached within 3 months rather than the 21 months planned. Without rapid identification, this would have meant that the research budget was being spent compensating bots, with 81.9% (4556/5562) of the final sample estimated to be bots. This would likely have resulted in an underestimation of the effectiveness of the intervention due to the noise from the bot responses.

We recommend using a CAPTCHA when setting up a remote trial to deter automated responses. We did not initially use one to ensure the trial was as accessible as possible and so as not to deter “real” participants. However, it is worth noting that a CAPTCHA does not render a survey invulnerable and could still be passed by a manual fraudulent entry [1].

Participant deception occurred on a smaller scale than the bots, accounting for 5% (298/5955) of participants enrolled, and as such, appears to be less of an issue. However, it is also less likely to be detected, and it is also more time intensive for researchers to try to identify suspicious responses based on the contact information provided. Establishing a strict procedure for identification and management meant the research team could act swiftly and deter future attempts. We suspect most of the participant deception was perpetrated by a relatively small number of individuals at different time points attempting to enroll on multiple occasions, as once several of their entries were removed with emails explaining why, the numbers engaging in deception decreased considerably. Without identification and management of this issue, individuals may have continued to submit false entries throughout the study.

There were 3 elements of the trial that helped the team to identify manual participant deception. First, participants were followed up 3 times over the course of 6 months, which presented several opportunities for researchers to contact participants and potentially identify any anomalies in contact details. With fewer resources and time spent on follow-up, some of the manual participant deception may have gone undetected. Second, there was no financial incentive offered for enrolling in the study initially, but only for completed follow-ups, so the incentive was delayed, making it less attractive for people seeking an immediate reward. Finally, the vouchers were sent manually by a member of the research team rather than being sent automatically, so there was a time lapse between follow-up completion and compensation being sent and a further opportunity to detect any discrepancies with the information given or similar or duplicate email addresses used before the vouchers were sent.

It is worth noting the potential issues with inequalities when creating data management procedures to detect participant deception and the need to strike a balance [23]. For example, not everyone has a mobile phone number to provide or a fixed address and is therefore unable to supply a valid home address. In this study, when this was found to be the case when contacting a participant, they remained in the study.

### Recommendations

Based on our experiences, we have made 6 recommendations for other researchers for limiting bots and manual participant deception:

1. Use CAPTCHAS.
2. Use attention checks.
3. Rigorous data management plan.
4. Be cautious with mentioning financial compensation in web-based advertising.
5. Consider the risk of introducing bias.
6. Plan for the additional resources required.

#### Use CAPTCHAs (and Other Available Automated Security Protections)

Ensure there are safeguards against automated bots when creating a web-based survey, particularly if financial compensation is involved. We used a CAPTCHA, also recommended by other studies [1] although in isolation, this is likely to be insufficient to identify manual participant deception. Investigate the tools available to protect against unwanted responses when selecting the survey platform and whether your institution has the appropriate license for their use. For example, there were additional security features Qualtrics [21] offers that may have helped with our problems but required additional cost and institutional permission and activation, as other studies have noted [8].

#### Use Attention Checks

Consider adding attention-check questions to the survey, which we found helpful. Examples include requiring participants to select a particular response option to a question, or use duplicate questions with absolute answers such as date of birth, and programming the survey to automatically flag respondents not providing matching responses [24].

#### Rigorous Data Management Plan

A detailed data management plan and data checking methods are important to protect the validity of data. It is a challenge for researchers to stay ahead of bots and manual participant deception and prevent them from completing surveys [23]. Each successful identification is another step toward less noisy and more accurate data and will help future researchers understand the many different methods available to safeguard research. Without rigorous data management, issues may not be noticed until later in recruitment (if at all), resulting in disruption of the study by bots and poor-quality data. This emphasizes the importance of thorough data management plans being established initially and the benefits of being reactive and adaptive to issues as they arise. For example, flexibility regarding advertising (recommendation 4).

#### Be Cautious With Mentioning Financial Compensation in Web-Based Advertising

Web-based advertising is often used in remote research to direct potential participants to surveys [1,4]. It is useful as participants can directly access the survey without having to type a link or scan a QR code.

However, advertising that financial compensation is available can invite participant deception, as in our case. A previous study investigated participation rates with and without financial incentives and reported that participation from nonunique IP addresses (so suspected duplication) was 6 times higher when an incentive was advertised compared to when it was not [12]. This has also been seen in other studies where being eligible for an incentive made it 6 times more likely that a participant would submit additional responses [25].

Response rate can be impacted by the amount of incentive, with an increase of US $5 resulting in a higher screener response rate recorded (29.9% vs 22.7%) [26]. Conversely, other studies reported no evidence that a larger financial incentive was associated with a higher rate of deception [27].

Other studies have reported no additional impact of the inclusion of an incentive compared to participants without an incentive [28]. Where financial compensation is deemed important for follow-up retention, we recommend either not mentioning the compensation or at least minimizing its prominence in web-based advertising. Reducing the prominence of the incentive helps keep traffic to the survey site limited to people who are genuinely interested in participating. We also recommend only providing financial compensation when follow-up questionnaires or tasks are completed, not at the point of sign-up, to deter those seeking a quick financial gain.

#### Consider the Risk of Introducing Bias

Compromises are required to protect against participant deception while trying to avoid deterring genuine participants [23]. Strategies to remove bots and false participants must be balanced with the risk of adding post-randomization bias to the study by removing genuine participants. We only removed participants verified as having provided false address information and who were not known at the phone number provided. Criteria for the identification and removal of fraudulent participants should be clear, rigorous, and agreed upon within the research team to reduce the risk of bias, as well as reactive if a new method of participant deception is identified. Neglecting to remove participants who provide false information can lead to problems with data integrity [12] and validity [3], so making the best judgment about whether a participant is genuine is crucial to protect this [1] and to minimize the inadvertent removal of genuine participants.

#### Plan for the Additional Resources Required

Consider the time and resources required for appropriate data management and the associated costs within project plans and funding applications, depending on the recruitment target and study length, as continuous monitoring and verification while data collection is ongoing is essential [8]. One of the more successful strategies employed to confirm identity was telephone contact. Genuine participants understood why we needed to call and verify their identity, and false numbers or details were straightforward to establish. However, this method is time intensive; in our highest recruiting month, we had over 600 participants enrolled, meaning approximately 15 hours were spent conducting address checks in addition to other tasks.

### Conclusions

Conducting research remotely has many advantages, but it is vulnerable to manual participant deception and automated bots posing as genuine participants, which can disrupt research and lead to low-quality data. At the outset of planning a remote study, we recommend using CAPTCHAs, using at least one attention check question in a screening or baseline survey, writing a rigorous data management plan, including dynamic protocols that can adapt in response to changes in deceit [10,11], being cautious with mentioning financial compensation, considering the risk of introducing bias when dealing with deception, and planning for the time and associated costs involved with monitoring recruitment deception when costing your study.

## Appendix

Multimedia Appendix 1 S1: Social media advert on Facebook and Twitter (1) (September-November 2020). S2: Social media advert on Facebook and Twitter (2) (financial compensation not mentioned) (December 2020 – March 2021). S3: Social media advert on Facebook and Twitter (3), financial compensation mentioned, amount unspecified (March-June 2021, and during other advertising periods).

Multimedia Appendix 2 Advert placed on NHS webpage.

Multimedia Appendix 3 Primary Care Poster advert.

Multimedia Appendix 4 Radio advert transcript.

Multimedia Appendix 5 Email to suspected bots.

Multimedia Appendix 6 Textbox S1. Email to suspected false participants, sent between October 7 and November 10, 2020 Textbox S2. Email to suspected false participants from November 11, 2020 Textbox S3. Email following spot checks where phone number was invalid or participant was not known.

Multimedia Appendix 7 CONSORT checklist.

## Abbreviations

CAPTCHA: Completely Automated Public Turing Test to Tell Computers and Humans Apart
CONSORT: Consolidated Standards of Reporting Trials
NHS: National Health Service
