# Supplementary material for: Bot or Not? Detecting and Managing Participant Deception When Conducting Digital Research Remotely: Case Study of a Randomized Controlled Trial
Source: J Med Internet Res. 2023 Sep 14;25:e46523. doi: 10.2196/46523 (PMC10540014; doi:10.2196/46523)

*Advertising used in different media*

**Figure S1**. Social media advert on Facebook and Twitter (1) (September-November 2020).


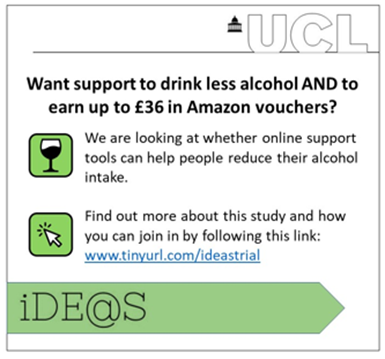


**Figure S2**.Social media advert on Facebook and Twitter (2) (financial compensation not mentioned) (December 2020 – March 2021).


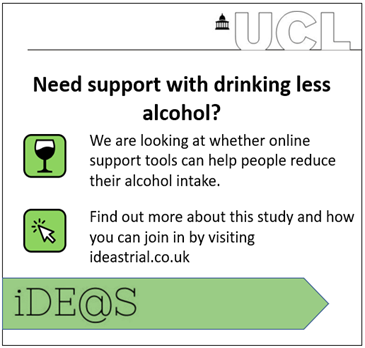


**Figure S3**. Social media advert on Facebook and Twitter (3), financial compensation mentioned, amount unspecified (March-June 2021, and during other advertising periods).


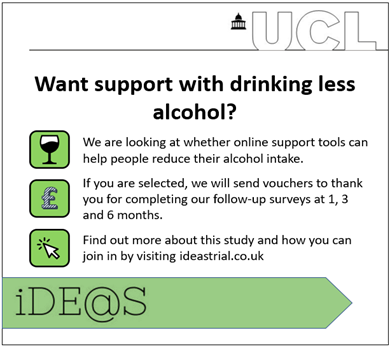

Supplement: Multimedia Appendix 1 [file jmir_v25i1e46523_app1.docx]
