# Supplementary figures and images for: Bot or Not? Detecting and Managing Participant Deception When Conducting Digital Research Remotely: Case Study of a Randomized Controlled Trial
Source: J Med Internet Res. 2023 Sep 14;25:e46523. doi: 10.2196/46523 (PMC10540014; doi:10.2196/46523)

*Appendix 2: Advert placed on NHS webpage*18


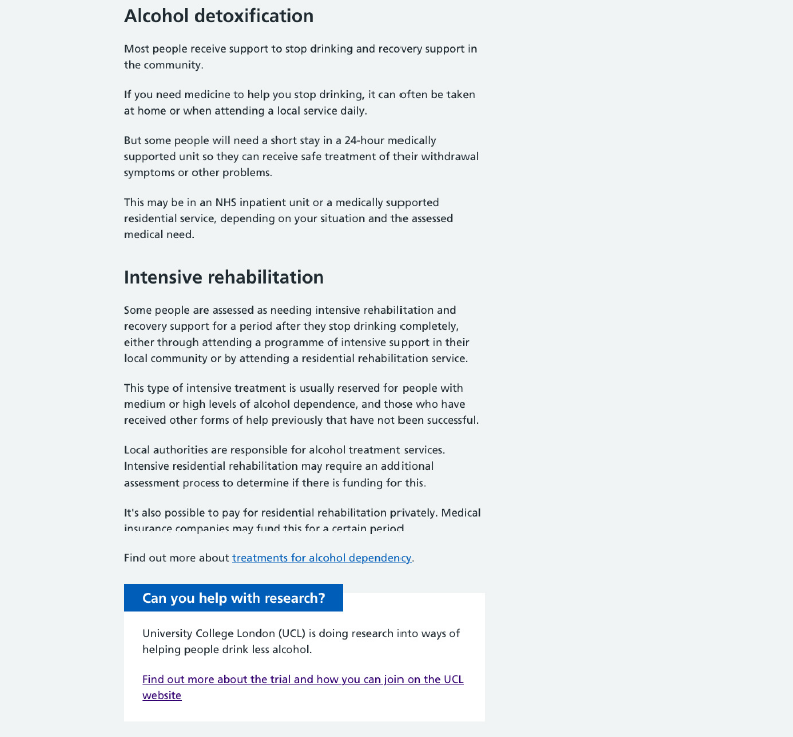

Supplement: Multimedia Appendix 2 [file jmir_v25i1e46523_app2.docx]

*Appendix 3:* Primary care poster


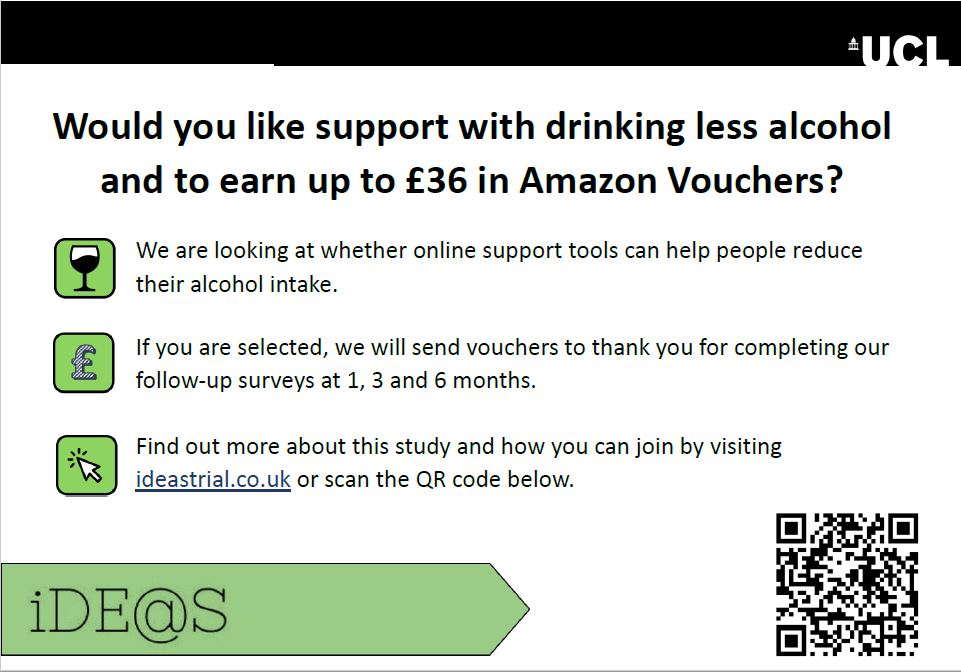

Supplement: Multimedia Appendix 3 [file jmir_v25i1e46523_app3.docx]
