# Supplementary material for: Bot or Not? Detecting and Managing Participant Deception When Conducting Digital Research Remotely: Case Study of a Randomized Controlled Trial
Source: J Med Internet Res. 2023 Sep 14;25:e46523. doi: 10.2196/46523 (PMC10540014; doi:10.2196/46523)
Supplement: Multimedia Appendix 4 [file jmir_v25i1e46523_app4.docx]

*Appendix 4: Radio advert transcript*

*“If you’re interested in reducing your alcohol consumption, then University College London need your help.*

*As part of our alcohol research study, we’re looking for participants to help evaluate the effectiveness of digital tools in helping people reduce their alcohol consumption.*

*If you’re interested in drinking less alcohol, and earning up to £36 in Amazon vouchers, find out if you’re eligible at Ideas Trial dot co dot UK.*

*That’s Ideas Trial dot co dot UK.”*
