# Supplementary material for: Bot or Not? Detecting and Managing Participant Deception When Conducting Digital Research Remotely: Case Study of a Randomized Controlled Trial
Source: J Med Internet Res. 2023 Sep 14;25:e46523. doi: 10.2196/46523 (PMC10540014; doi:10.2196/46523)
Supplement: Multimedia Appendix 5 [file jmir_v25i1e46523_app5.docx]

*Appendix 5: Email to suspected bots*

*Subject: Withdrawal from iDEAS trial*

Dear [FirstName],

We have identified issues with the responses you provided to the iDEAS screening survey and have therefore withdrawn you as a participant. If you believe that we have made a mistake please reply to this email ([m.oldham@ucl.ac.uk](mailto:m.oldham@ucl.ac.uk)) within 24 hours and we will call you back on the number you provided at registration to confirm some details.

Kind regards,
