# Supplementary material for: Bot or Not? Detecting and Managing Participant Deception When Conducting Digital Research Remotely: Case Study of a Randomized Controlled Trial
Source: J Med Internet Res. 2023 Sep 14;25:e46523. doi: 10.2196/46523 (PMC10540014; doi:10.2196/46523)
Supplement: Multimedia Appendix 6 [file jmir_v25i1e46523_app6.docx]

**Textbox S1**. Email to suspected false participants, sent between October 7 and November 10, 2020.

Subject: Withdrawal from iDEAS trial

Dear [FirstName],

We have identified issues with the responses you provided to the iDEAS screening survey and have therefore withdrawn you as a participant. If you believe that we have made a mistake please reply to this email ([m.oldham@ucl.ac.uk](mailto:m.oldham@ucl.ac.uk)) within 24 hours and we will call you back on the number you provided at registration to confirm some details.

Kind regards,

**Textbox S2**. Email to suspected false participants from November 11, 2020.

Subject: Withdrawal from iDEAS trial

Dear [FirstName],

We have identified issues with the data you provided to the iDEAS screening survey and have therefore withdrawn you as a participant.

If you feel that we have made a mistake please email me on [gemma.loebenberg@ucl.ac.uk](mailto:gemma.loebenberg@ucl.ac.uk) within 72 hours and I will call you back on the number you provided at registration. If I do not hear from you within 72 hours I will consider this matter resolved.

Best wishes

**Textbox S3**. Email following spot checks where phone number was invalid or participant was not known.

*Subject: Confirmation for iDEAS trial*

Dear [FirstName]

I have just tried to call you on the number you provided when you signed up to the iDEAS trial, and I could not reach you at this number.

It is necessary that you provide a contact telephone number as part of the enrolment for the study.

We need to speak to you to confirm your details by (insert date); please could you provide me with an alternative phone number to speak to you on?  Alternatively please call me on 020 7679 8781.

If we do not hear from you by this time, we will have to remove you from the study and will be unable compensate you for any future follow-up surveys.

We look forward to speaking with you.
