# Supplementary material for: Bot or Not? Detecting and Managing Participant Deception When Conducting Digital Research Remotely: Case Study of a Randomized Controlled Trial
Source: J Med Internet Res. 2023 Sep 14;25:e46523. doi: 10.2196/46523 (PMC10540014; doi:10.2196/46523)
Supplement: Multimedia Appendix 7 [file jmir_v25i1e46523_app7.pdf]

Reporting checklist for randomised trial.

Based on the CONSORT guidelines.

Instructions to authors

Complete this checklist by entering the page numbers from your manuscript where readers will find each of the items listed below.

Your article may not currently address all the items on the checklist. Please modify your text to include the missing information. If you are certain that an item does not apply, please write "n/a" and provide a short explanation.

Upload your completed checklist as an extra file when you submit to a journal.

In your methods section, say that you used the CONSORTreporting guidelines, and cite them as:

Schulz KF, Altman DG, Moher D, for the CONSORT Group. CONSORT 2010 Statement: updated guidelines for reporting parallel group randomised trials

		Reporting Item	Page Number
**Title and Abstract**			
Title	#1a	Identification as a randomized trial in the title.	Pg 2
Abstract	#1b	Structured summary of trial design, methods, results, and conclusions	Pg 2
**Introduction**			
Background and objectives	#2a	Scientific background and explanation of rationale	Pg 3
Background and objectives	#2b	Specific objectives or hypothesis	Pg 4
**Methods**			
Trial design	#3a	Description of trial design (such as parallel, factorial)	n/a this paper is not

		including allocation ratio.	discussing the trial, rather issues that arose during recruitment
Trial design	#3b	Important changes to methods after trial commencement (such as eligibility criteria), with reasons	n/a there were no changes to trial methods, there were changes to recruitment methods, described on pgs 5-7
Participants	#4a	Eligibility criteria for participants	n/a
Participants	#4b	Settings and locations where the data were collected	4
Interventions	#5	The experimental and control interventions for each group with sufficient details to allow replication, including how and when they were actually administered	n/a this will be described in the main trial paper
Outcomes	#6a	Completely defined prespecified primary and secondary outcome measures, including how and when they were assessed	n/a these will be described in the main trial paper
Outcomes	#6b	Any changes to trial outcomes after the trial commenced, with reasons	n/a to this paper
Sample size	#7a	How sample size was	Will be

		determined.		described in main trial paper
Sample size	#7b	When applicable, explanation of any interim analyses and stopping guidelines		Will be described in main trial paper
Randomization - Sequence generation	#8a	Method used to generate the random allocation sequence.	Will be described in main trial paper	
Randomization - Sequence generation	#8b	Type of randomization; details of any restriction (such as blocking and block size)	Will be described in main trial paper	
Randomization - Allocation concealment mechanism	#9	Mechanism used to implement the random allocation sequence (such as sequentially numbered containers), describing any steps taken to conceal the sequence until interventions were assigned		Will be described in main trial paper
Randomization - Implementation	#10	Who generated the allocation sequence, who enrolled participants, and who assigned participants to interventions		Will be described in main trial paper
Blinding	#11a	If done, who was blinded after assignment to interventions (for example, participants, care providers,		Will be described in main trial paper

		those assessing outcomes) and how.		
Blinding	#11b	If relevant, description of the similarity of interventions	Will be described in main trial paper	
Statistical methods	#12a	Statistical methods used to compare groups for primary and secondary outcomes		Will be described in main trial paper
Statistical methods	#12b	Methods for additional analyses, such as subgroup analyses and adjusted analyses		Will be described in main trial paper
**Results**				
Participant flow diagram (strongly recommended)	#13a	For each group, the numbers of participants who were randomly assigned, received intended treatment, and were analysed for the primary outcome		Enrolment diagram on p5
Participant flow	#13b	For each group, losses and exclusions after randomization, together with reason		Exclusions detailed pgs 5-10
Recruitment	#14a	Dates defining the periods of recruitment and follow-up		Pg 4
Recruitment	#14b	Why the trial ended or was stopped		n/a
Baseline data	#15	A table showing baseline demographic and clinical characteristics for each group		n/a will be described in main paper
Numbers	#16	For each group, number of		n/a will be

analysed		participants (denominator) included in each analysis and whether the analysis was by original assigned groups	described in main paper
Outcomes and estimation	#17a	For each primary and secondary outcome, results for each group, and the estimated effect size and its precision (such as 95% confidence interval)	n/a Will be described in main trial paper
Outcomes and estimation	#17b	For binary outcomes, presentation of both absolute and relative effect sizes is recommended	n/a will be described in main paper
Ancillary analyses	#18	Results of any other analyses performed, including subgroup analyses and adjusted analyses, distinguishing pre-specified from exploratory	n/a will be described in main paper
Harms	#19	All important harms or unintended effects in each group (For specific guidance see CONSORT for harms)	n/a
**Discussion**			
Limitations	#20	Trial limitations, addressing sources of potential bias, imprecision, and, if relevant, multiplicity of analyses	Pg 11
Generalisability	#21	Generalisability (external validity, applicability) of the trial findings	Pg 11-13
Interpretation	#22	Interpretation consistent with results, balancing benefits	Pg 11-13

		and harms, and considering other relevant evidence	
Registration	#23	Registration number and name of trial registry	Pg 14
**Other information**			
Interpretation	#22	Interpretation consistent with results, balancing benefits and harms, and considering other relevant evidence	n/a
Registration	#23	Registration number and name of trial registry	Pg 14
Protocol	#24	Where the full trial protocol can be accessed, if available	Pg 22
Funding	#25	Sources of funding and other support (such as supply of drugs), role of funders	Pg 14

None The CONSORT checklist is distributed under the terms of the Creative Commons Attribution License CC-BY. This checklist can be completed online using https://www.goodreports.org/, a tool made by the EQUATOR Network in collaboration with Penelope.ai
